# Insulin-like growth factor levels in cord blood, birth weight and breast cancer risk

**DOI:** 10.1038/sj.bjc.6605074

**Published:** 2009-05-05

**Authors:** P Lagiou, C C Hsieh, L Lipworth, E Samoli, W Okulicz, R Troisi, B Xu, P Hall, A Ekbom, H O Adami, D Trichopoulos

**Affiliations:** 1 Department of Epidemiology, Harvard School of Public Health, 677 Huntington Avenue, Boston, MA 02115, USA; 2 Department of Hygiene, Epidemiology and Medical Statistics, School of Medicine, University of Athens, 75M. Asias Street, Athens GR-115 27, Greece; 3 Department of Medical Epidemiology and Biostatistics, Box 281, Karolinska Institutet, Stockholm SE-171 77, Sweden; 4 Division of Biostatistics and Epidemiology, UMass Cancer Center, University of Massachusetts Medical School, 364 Plantation Street, LRB 427, Worcester, MA 01605, USA; 5 International Epidemiology Institute, 1455 Research Blvd, Suite 550, Rockville, MD 20850, USA; 6 Department of Physiology, ILAT Steroid RIA Laboratory, University of Massachusetts Medical School, Worcester, MA 01605, USA; 7 Division of Cancer Epidemiology and Genetics, Department of Health and Human Services, National Cancer Institute, National Institutes of Health,6120 Executive Boulevard, MSC 7242, Bethesda, MD 20892-7242, USA; 8 Department of Epidemiology, School of Public Health, Fudan University, Shanghai 200023, China; 9 Clinical Epidemiology Unit, Department of Medicine, Karolinska Institutet/Karolinska University Hospital, Stockholm SE-171 76, Sweden

**Keywords:** insulin-like growth factor, IGF-1, IGF-2, breast cancer, birth weight, birth length

## Abstract

Breast cancer incidence and birth weight are higher among Caucasian than Asian women, and birth size has been positively associated with breast cancer risk. Pregnancy hormone levels, however, have been generally lower in Caucasian than Asian women. We studied components of the insulin-like growth factor (IGF) system in cord blood from 92 singleton babies born in Boston, USA, and 110 born in Shanghai, China, in 1994–1995. Cord blood IGF-1 was significantly higher among Caucasian compared with Chinese babies (*P*<10^−6^). The opposite was noted for IGF-2 (*P∼*10^−4^). IGF-1 was significantly positively associated with birth weight and birth length in Boston, but not Shanghai. In contrast, stronger positive, though statistically non-significant, associations of IGF-2 with birth size were only evident in Shanghai. The associations of birth weight and birth length were positive and significant in taller women (for IGF-1 in Boston *P∼*0.003 and 0.03, respectively; for IGF-2 in Shanghai *P∼*0.05 and ∼0.04, respectively), among whom maternal anthropometry does not exercise strong constraints in foetal growth. The documentation of higher cord blood levels of IGF-1, a principal growth hormone that does not cross the placenta, among Caucasian than in Asian newborns is concordant with breast cancer incidence in these populations.

During the past few decades, the possibility has been investigated that breast cancer can have roots in early life, including the intrauterine period. By the late 2000s, birth size had become a predictor of breast cancer risk (Michels and Xue, 2006; Park *et al*, 2008; dos Santos Silva *et al*, 2008). We have hypothesised that high levels of mammotropic hormones during pregnancy favour the generation of mammary-tissue-specific stem cells in the offspring and that the pool of these cells is an important predictor of risk (Trichopoulos *et al*, 2005). Because such stem cells are difficult to isolate, haematopoietic stem and progenitor cells have been used as markers. Concentration of these cells in cord blood is strongly positively associated with both cord blood levels of insulin-like growth factor 1 (IGF-1) (Savarese *et al*, 2007) and birth weight (Strohsnitter *et al*, 2008), suggesting that IGF-1 may be an important factor in the intrauterine origin of breast cancer.

The incidence of breast cancer (Ferlay *et al*, 2004), as well as birth weight (Wen *et al*, 1995; Lagiou *et al*, 2003), are higher among Caucasian women in western countries than in Asian women in the east. We hypothesised, therefore, that cord blood levels of IGF-1, which does not cross the placenta (Holmes *et al*, 1999), are higher among Caucasian than among Asian neonates, and that cord blood IGF-1 levels are positively associated with birth size. We evaluated this hypothesis, and also examined the role of IGF-2, a main component of the IGF system in foetal life, by studying cord blood samples from babies born to women in Boston, USA and Shanghai, China.

## Materials and methods

Study participants were adult pregnant women and their offspring, recruited from maternity clinics affiliated with two centres: Beth Israel Hospital in Boston, USA, and Shanghai Medical University in China. The study was approved by the Institutional Review Boards of the two centres, as well as the Institutional Review Boards of the Harvard School of Public Health and the US Department of the Army. Details on the study have been reported earlier (Lipworth *et al*, 1999) and are summarised below.

At each centre, an authorised health professional met all pregnant women coming for their first routine prenatal visit to the collaborating maternity clinic, ascertained whether the woman was eligible to participate, explained to her the objectives of the study and the requirements for participation, and obtained informed consent. To be eligible, a pregnant woman had to be < 40 years of age, have no more than one previous (liveborn or stillborn) child, be Caucasian in Boston and Chinese in Shanghai, and be able to understand and speak the local language. Women were excluded if they had taken any hormonal medication during the index pregnancy, if they had an earlier diagnosis of diabetes mellitus or thyroid disease, or if the foetus had a known major anomaly.

Between March 1994 and October 1995, among 402 women identified at Beth Israel Hospital in Boston, 304 pregnant women agreed to participate and met the eligibility criteria. In Shanghai, among 424 women identified between April 1994 and May 1995, 334 pregnant women agreed to participate and met the eligibility criteria. In both centres, gestational age was defined as the time since the first day of the last menstrual period. Baseline sociodemographic information was recorded and blood was drawn at gestational weeks 16 and 27. Cord blood was collected and additional information concerning the delivery and the newborn was ascertained from medical records and paediatric charts.

Cord blood was collected in sterile tubes without preservatives and refrigerated at 4°C for up to 24 h until centrifugation, after which, serum from each sampling was separated and distributed into aliquots. In Shanghai, cord blood samples were transported in a cooler to a laboratory near Shanghai Medical University. Serum aliquots were stored at −20°C for 5–7 days in the laboratory before being transported to Shanghai Medical University and stored at −80°C. All samples were shipped by air on dry ice to Boston where they were stored at −80°C together with the cord blood samples from Boston. We were able to collect cord blood samples of acceptable quality and sufficient quantities for the determinations of cord blood hormones for 202 uncomplicated full-term pregnancies (37–42 weeks long without pregnancy toxaemia), 92 in Boston and 110 in Shanghai.

Hormone determinations were conducted at the ILAT Steroid RIA Laboratory of the University of Massachusetts Medical School. IGF-1, IGF-2, and IGFBP-3 were measured by coated-tube immunoradiometric assay kits (Diagnostic System Laboratories, Inc., Webster, TX, USA). The laboratory-estimated inter-assay and intra-assay coefficients of variation were, respectively, 9.0% and 3.3% for IGF-1, 5.9% and 3.4% for IGF-2, and 8.0% and 4.8% for IGFBP-3. There was no detectable cross reactivity of the IGF-1 assay with IGF-2 according to the manufacturer's specificity assessment.

### Statistical analyses

Statistical analyses were conducted using the SPSS statistical package (Statistical Package for Social Sciences v. 16, Chicago, IL, USA). Multiple regression models were used to compare hormone levels between Boston and Shanghai controlling for maternal age, height, duration of gestation and weight gain (all continuously), as well as for parity and gender of offspring. The association of IGF-1 and IGF-2 with birth weight and birth length were examined by modelling the data through multiple regression models with, alternatively, birth weight and length as the outcomes, controlling also mutually for IGF-1 and IGF-2, as well as for IGFBP-3. Analyses were conducted separately for each centre, for all women, as well as for tall and short women, with the cut-off for height set *a priori* at 163 cm (median height in Boston, third quartile in Shanghai) based on the results of an earlier study (Lagiou *et al*, 2005). The hypothesis is that, among shorter women, maternal anthropometry imposes constraints on birth size (Wen *et al*, 1995; Lagiou *et al*, 2003) and could trigger negative feedback mechanisms that could obscure positive associations of hormones with birth size.

## Results

Characteristics of mothers and offspring and cord blood levels of IGF-1, IGF-2, and IGFBP-3 are shown in Table 1. Caucasian mothers in Boston were significantly older and taller than Chinese mothers in Shanghai. In Boston, mothers were in almost equal proportions primi- and biparae, whereas in Shanghai virtually all were primiparae. The number of boys and girls was identical in Boston, but there were substantially more boys than girls in Shanghai. Birth weight and length were higher in Boston. Table 1 also shows unadjusted mean values and standard deviations of the three studied components of the IGF axis. Cord blood levels of IGF-1 were higher in Boston than in Shanghai, whereas the opposite was evident with respect to IGF-2, while IGFBP-3 levels were higher in Shanghai.

In Table 2, cord blood levels of IGF-1, IGF-2, and IGFBP-3 are compared between cities adjusting for maternal age, height, weight gain, parity, duration of gestation, and gender of offspring. Levels of IGF-1 were significantly and substantially higher in Boston, whereas IGF-2 levels were significantly lower. The differences in IGF-1 and IGF-2 were amplified after adjustment both mutually as well as for IGFBP-3. As essentially all women in Shanghai were primiparae, we repeated the analyses for primiparae women only and the results were essentially unchanged.

Table 3 shows multiple regression-derived partial regression coefficients of birth weight (upper panel) and birth length (lower panel) on one standard deviation increments of cord blood levels of IGF-1 and IGF-2 in Boston and Shanghai, both overall and in strata defined by maternal height. When all women within each centre were studied, results were adjusted for maternal age, height, parity, weight gain, duration of gestation, and gender of offspring. In the stratified analyses, in which the number of observations was considerably reduced, parity and offspring gender, which minimally affected within-centre estimates, were not included among the covariates. In all models, IGF-1 and IGF-2 were adjusted both mutually and for IGFBP-3. In Boston, with respect to both birth weight and birth length, there were significant positive associations with IGF-1; stratified analyses indicated that these differences were generated exclusively by the offspring of taller women. In contrast, in Shanghai, IGF-1 was suggestively positively associated only with birth length and only among taller women (*P∼*0.11).

The results for IGF-2 were strikingly different. In Boston, no association was evident with respect to either birth weight or birth length, neither among women overall nor among taller or shorter women. In Shanghai, however, cord blood IGF-2 was suggestively positively associated with birth length (*P∼*0.09), whereas, among taller women, it was significantly positively associated with both birth weight and length. The cut-off of 163 cm for maternal height was set *a priori*. Nevertheless, with respect to birth weight, the *P*-value for interaction of maternal height with IGF-1 (both continuously) was 0.09 for Boston, whereas the *P* for interaction of maternal height with IGF-2 was 0.01 in Shanghai.

## Discussion

In our study involving pregnancies of 110 Asian women in China and 92 Caucasian women in USA, we have found that cord blood IGF-1 was significantly higher in Boston compared with Shanghai (*P*<10^−6^), whereas the opposite was noted with respect to IGF-2 (*P∼*10^−4^) (Table 2). IGF-1 was positively associated with both birth weight and birth length among newborns in Boston, but not in Shanghai. With respect to IGF-2 in relation to birth size, there were suggestive positive associations in Shanghai mostly with respect to birth length (*P*∼0.09) (Table 3).

In an earlier study (Lagiou *et al*, 2005), we evaluated the association of pregnancy estriol in maternal sera with birth weight after stratification of women by stature. The results supported our hypothesis that, because among shorter women maternal anthropometry imposes stronger constraints on birth size (Wen *et al*, 1995; Lagiou *et al*, 2003), negative feedback mechanisms might be triggered that masked positive associations of hormones with birth size. Similarly, in this study, the positive associations of cord blood IGF with birth weight and length were significant among taller women for IGF-1 in Boston and for IGF-2 in Shanghai, and essentially null among women of shorter stature.

Strengths of this investigation are the inclusion of participants from two populations with contrasting incidence of breast cancer, the implementation of a uniform protocol, the use of state of-the-art assays in a qualified laboratory, and the appreciable study size for a study of this nature. Limitations include lack of measurements of other IGF-binding proteins and IGF receptors.

Birth size is positively associated with breast cancer risk several decades later (Michels and Xue, 2006; Park *et al*, 2008; dos Santos Silva *et al*, 2008) and birth weight is higher among Caucasian newborns in the United States compared with Asian newborns in China (Wen *et al*, 1995; Lagiou *et al*, 2003). The documentation of higher cord blood levels of IGF-1 among Caucasian compared with Asian newborns is concordant with the higher incidence of breast cancer in western compared with eastern Asian populations (Ferlay *et al*, 2004) and compatible with the role IGF appears to play in breast cancer, at least among premenopausal women (Renehan *et al*, 2004; Schernhammer *et al*, 2005). Of note, IGF-1 does not cross the placenta (Holmes *et al*, 1999) and this was also supported by our data, in which the correlation coefficients between cord blood IGF-1 and maternal IGF-1 were very low. Our finding of higher cord blood levels of IGF-1 among Caucasian compared with Asian newborns is of particular importance because, in the same dataset, maternal pregnancy estradiol and estriol (Lipworth *et al*, 1999), as well as cord blood estriol, androstenedione, and testosterone (Troisi *et al*, 2008) have been reported to be significantly higher among Chinese than among Caucasian women. In a small subsample of 52 US and 22 Chinese newborns from the same dataset, in which IGF-2 was not measured, there was no significant difference between the two groups with respect to cord blood IGF-1 and, possibly by chance owing to the small subsample size, levels of IGF-1 appeared to be somewhat higher among Chinese newborns (Troisi *et al*, 2008).

Our results indicating that IGF-1 dominates foetal growth among Caucasians, whereas IGF-2 plays a similar role among Asians and that associations are evident among taller mothers are not directly comparable with previous studies, because mutual adjustment of IGF-1 and IGF-2 and stratification by maternal height were not generally undertaken. Nevertheless, cord blood IGF-1 has shown a consistently positive association with birth weight in Caucasian (Gluckman *et al*, 1983; Ashton *et al*, 1985; Ostlund *et al*, 1997; Ong *et al*, 2000; Christou *et al*, 2001) and less consistently in Asian populations (Wang *et al*, 1991; Yang and Kim, 2000; Yang and Yu, 2000; Lo *et al*, 2002; Hung *et al*, 2008). Cord blood IGF-2 associations with birth weight are generally weakly positive or null among both Caucasians (Gluckman *et al*, 1983; Ashton *et al*, 1985; Ong *et al*, 2000) and Asians (Lo *et al*, 2002; Pathmaperuma *et al*, 2007; Hung *et al*, 2008).

Cord blood IGF-1 has been shown to be positively associated with the size of the stem cell pool (Baik *et al*, 2005; Savarese *et al*, 2007), which has also been linked to birth size (Strohsnitter *et al*, 2008). The stem cell pool has been postulated to be related to breast cancer risk in later life (Trichopoulos *et al*, 2005). We found no reports concerning a possible association of this pool with IGF-2, which is a growth promoting hormone during gestation (O’Dell and Day, 1998). In the few analyses based on Asian populations, there was no relationship between birth size and breast cancer risk in later life (dos Santos Silva *et al*, 2008). The differential actions of IGF-1 and IGF-2 in embryonic life could be explained by the fact that both IGFs are known to bind to the signalling IGF-1 receptor, whereas IGF-2 also binds to the non-signalling IGF-2 receptor (Ong *et al*, 2000).

Irrespectively of the underlying physiologic mechanisms, the difference in birth size between Caucasian and Asian newborn can account for only a small fraction of the differences in breast cancer incidence. However, the fact that endocrine perinatal influences on birth size are evident mostly, or exclusively, among newborn of taller women (Table 3) may explain the sharp contrast in breast cancer incidence between Caucasian and Asian women—birth size is positively associated with adult height (Michels *et al*, 2006) and, over successive generations, improved nutrition, leading to increased adult body size, might reduce constraints on foetal growth and birth size (Wen *et al*, 1995; Lagiou *et al*, 2003), which in turn affects adult height. The cycle tends to repeat itself, notably over consecutive generations of Asians migrating to the west, who show a gradual increase of breast cancer incidence (Lagiou *et al*, 2003; Lagiou and Trichopoulos, 2008). Changes in age at first pregnancy, parity, and lactation also play a role in the increases of breast cancer among Asian migrants to western countries (Haenszel and Kurihara, 1968; Buell, 1973; Ziegler *et al*, 1993).

## Figures and Tables

**Table 1:** Characteristics^a^ of mothers and their offspring and cord blood levels of IGF-1, IGF-2, and IGFBP-3

	**Boston (*n*=92)**	**Shanghai (*n*=110)**
Age (years)	31.0 (3.0)	25.1 (3.3)
Maternal height (cm)	164.1 (7.2)	160.2 (4.9)
		
*Maternal height*		
⩽1.63 m	46 (50.0%)	81 (73.6%)
>1.63 m	46 (50.0%)	29 (26.4%)
		
*Parity*		
1	48 (52.2%)	109 (99.1%)
2	44 (47.8%)	1 (0.9%)
		
Duration of gestation (weeks)	40.1 (1.1)	40.0 (1.1)
Maternal weight gain (kg)^b^	11.5 (3.9)	8.9 (4.5)
		
*Gender of offspring*		
Male	46 (50.0%)	64 (58.2%)
Female	46 (50.0%)	46 (41.8%)
Birth weight (g)	3557.7 (490.2)	3492.6 (459.8)
Birth length (cm)	50.6 (2.5)	49.8 (3.1)
		
IGF-1 (ng ml^−1^)	98.4 (37.8)	79.0 (48.9)
IGF-2 (ng ml^−1^)	492.5 (100.3)	587.8 (140.1)
IGFBP-3 (ng ml^−1^)	2419.1 (1696.8)	3265.3 (2186.4)

IGF=insulin-like growth factor.

Uncomplicated full-term singleton pregnancies in Boston, USA and Shanghai, China.

a For continuous variables mean (s.d.); for categorical variables *n* (%).

b Until the 27th week of gestation.

**Table 2:** Percent differences^a^ of cord blood levels of IGF-1, IGF-2, and IGFBP-3 between newborns in Boston, USA (reference) and Shanghai, China

	**Unadjusted for the other IGF components**		**Adjusted for the other IGF components**	
	**Shanghai *vs* Boston (%)**	***P*-value**	**Shanghai *vs* Boston (%)**	***P*-value**
IGF-1	−39.6 (−54.3, −20.2)	0.0005	−50.9 (−61.0, −38.1)	<10^−6^
IGF-2	20.7 (7.7, 35.2)	0.001	23.2 (10.9, 37.0)	0.0001
IGFBP-3	23.0 (−8.1, 64.7)	0.162	22.5 (−5.1, 57.9)	0.118

IGF=insulin-like growth factor.

a Adjusted for maternal age, height and weight gain, parity, duration of gestation, and gender of offspring. Hormone levels were log-transformed, so that the coefficients express percentage differences between centres.

**Table 3:** Multiple regression-derived partial regression coefficients b^a^ (and 95% confidence intervals, CIs) of birth weight (upper panel) and birth length (lower panel) on one standard deviation increments of cord blood levels of IGF-1, IGF-2, and IGFBP-3 in Boston, USA and Shanghai, China, overall and by maternal height. Statistically significant *P* values (<0.05) are indicated in bold fonts.

	**All women**				**Women ⩽1.63 m height**				**Women >1.63 m height**			
	**Boston (*n*=92)**		**Shanghai (*n*=110)**		**Boston (*n*=46)**		**Shanghai (*n*=81)**		**Boston (*n*=46)**		**Shanghai (*n*=29)**	
	***b* (CI)**	***P*-value**	***b* (CI)**	***P*-value**	***b* (CI)**	***P*-value**	***b* (CI)**	***P*-value**	***b* (CI)**	***P*-value**	***b* (CI)**	***P*-value**
*Birth weight*												
IGF-1 (per 44.4 ng ml^−1^)	141.7 (11.3, 272.1)	**0.03**	−3.7 (−133.5, 126.0)	0.96	−39.6 (−252.3, 173.1)	0.71	−1.2 (−139.9, 137.4)	0.99	260.4 (92.6, 428.1)	**0.003**	−33.6 (−420.6, 353.3)	0.86
IGF−2 (per 132.1 ng ml^−1^)	7.0 (−131.4, 145.3)	0.92	57.3 (−51.3, 166.0)	0.30	45.5 (−137.2, 228.1)	0.62	−2.3 (−124.1, 119.5)	0.97	1.0 (−196.7, 198.7)	0.99	292.8 (0.59, 584.9)	**0.05**
												
*Birth length*												
IGF-1 (per 44.4 ng ml^−1^)	0.85 (0.19, 1.51)	**0.01**	0.00 (−0.91, 0.91)	0.99	0.29 (−0.70, 1.28)	0.56	−0.21 (−1.39, 0.98)	0.73	1.14 (0.09, 2.19)	**0.03**	0.81 (−0.19, 1.81)	0.11
IGF-2 (per 132.1 ng ml^−1^)	0.10 (−0.60, 0.80)	0.77	0.65 (−0.11, 1.41)	0.09	0.44 (−0.41, 1.29)	0.30	0.73 (−0.31, 1.77)	0.17	−0.17 (−1.41, 1.06)	0.78	0.78 (0.02, 1.53)	**0.04**

IGF=insulin-like growth factor; CI=confidence interval.

a Adjusted for maternal age, height and weight gain, parity, duration of gestation, and gender of offspring in models for all women; adjusted for maternal age and weight gain, and duration of gestation in models by maternal height. In all models, IGF-1 and IGF-2 were adjusted both mutually and for IGFBP-3.
